# Esophagitis and Pneumonitis Related to Concurrent Chemoradiation ± Durvalumab Consolidation in Unresectable Stage III Non-Small-Cell Lung Cancer: Risk Assessment and Management Recommendations Based on a Modified Delphi Process

**DOI:** 10.3390/curroncol31110483

**Published:** 2024-10-23

**Authors:** Anthony M. Brade, Houda Bahig, Andrea Bezjak, Rosalyn A. Juergens, Charmaine Lynden, Nicolas Marcoux, Barbara Melosky, Devin Schellenberg, Stephanie Snow

**Affiliations:** 1 Trillium Health Partners, Mississauga, ON L5B 1B8, Canada; 2 Department of Radiation Oncology, Peel Regional Cancer Centre, Mississauga, ON L5M 7S4, Canada; 3 Department of Radiation Oncology, University of Toronto, Toronto, ON M5T 1P5, Canada; 4 Department of Radiation Oncology, Centre Hospitalier de l’Université de Montréal, Montréal, QC H2X 0C1, Canada; 5 Radiation Medicine Program, Princess Margaret Cancer Centre, University Health Network, Toronto, ON M5G 2M9, Canada; 6 Division of Medical Oncology, McMaster University, Juravinski Cancer Centre, Hamilton, ON L8V 5C2, Canada; 7 Division of Hematology and Oncology, CHU de Québec, Québec City, QC G1R 2J6, Canada; 8 Department of Medical Oncology, BC Cancer, Vancouver, BC V5Z 4E6, Canada; 9 Department of Radiation Oncology, BC Cancer, Surrey, BC V3V 1Z2, Canada; 10 Division of Medical Oncology, Dalhousie University, Queen Elizabeth II Health Sciences Centre, Halifax, NS B3H 1V8, Canada

**Keywords:** lung cancer, concurrent chemoradiation therapy, immuno-oncology, esophagitis, pneumonitis, durvalumab, recommendations, management guidelines, supportive care

## Abstract

The addition of durvalumab consolidation to concurrent chemoradiation therapy (cCRT) has fundamentally changed the standard of care for patients with unresectable stage III non-small-cell lung cancer (NSCLC). Nevertheless, concerns related to esophagitis and pneumonitis potentially impact the broad application of all regimen components. A Canadian expert working group (EWG) was convened to provide guidance to healthcare professionals (HCPs) managing these adverse events (AEs) and to help optimize the patient experience. Integrating literature review findings and real-world clinical experience, the EWG used a modified Delphi process to develop 12 clinical questions, 30 recommendations, and a risk-stratification guide. The recommendations address risk factors associated with developing esophagitis and pneumonitis, approaches to risk mitigation and optimal management, and considerations related to initiation and re-initiation of durvalumab consolidation therapy. For both AEs, the EWG emphasized the importance of upfront risk assessment to inform the treatment approach, integration of preventative measures, and prompt initiation of suitable therapy in alignment with AE grade. The EWG also underscored the need for timely, effective communication between multidisciplinary team members and clarity on responsibilities. These recommendations will help support HCP decision-making related to esophagitis and pneumonitis arising from cCRT ± durvalumab and improve outcomes for patients with unresectable stage III NSCLC.

## 1. Introduction

Lung cancer remains the most commonly diagnosed malignancy and leading cause of cancer-associated mortality worldwide [1]. Among patients with unresectable stage III non-small-cell lung cancer (NSCLC), concurrent chemoradiation therapy (cCRT) is the standard of care (SoC). In 2017, the results of the phase III PACIFIC trial established the role of adding durvalumab, a programmed cell death ligand 1 (PD-L1) inhibitor, as consolidation therapy after cCRT [2,3]. The trial showed that addition of up to 12 months of the immuno-oncology (IO) therapy, initiated 1 to 42 days after cCRT completion, significantly improved progression-free survival and overall survival (OS) [2,3]. Durvalumab consolidation was quickly adopted into clinical practice and is now internationally recommended for patients with unresectable stage III NSCLC who are eligible for definitive cCRT [4,5,6,7,8]. The therapy has also recently shown benefit when used as consolidation in limited-stage small-cell lung cancer (LS-SCLC) [9].

Despite the demonstrated efficacy and guideline-recommended use of cCRT ± durvalumab consolidation for unresectable stage III NSCLC, several studies report that a disproportionately small number of eligible patients receive curative-intent cCRT [10,11,12]. Furthermore, use of durvalumab consolidation therapy may also be suboptimal [13,14]. Numerous factors may contribute to limited use of cCRT ± durvalumab [15], such as challenges related to drug access and treatment planning and delivery, multidisciplinary team (MDT) availability, communication, and collaboration, as well as patient suitability and concerns regarding toxicities and monitoring. Of the toxicities associated with cCRT ± durvalumab, esophagitis and pneumonitis are particularly frequent and challenging-to-manage adverse events (AEs) associated with potentially severe sequelae (Table 1) [16]. In meta-analyses conducted by Palma and colleagues, rates of symptomatic (grade ≥ 2; Table 2) esophagitis and pneumonitis were approximately 50% and 30%, respectively, after cCRT [17,18]. In the PACIFIC trial, any-grade pneumonitis (all etiologies) was experienced by 33.9% of durvalumab-treated patients (24.8% of placebo group) [2]. Although esophagitis was observed in only a small proportion of patients in the durvalumab arm (0.2% vs. 0% with placebo; only serious cases reported), AE evaluation likely occurred after most cases had resolved. Across real-world studies, frequencies of both esophagitis and pneumonitis vary considerably on the basis of patient and treatment characteristics [19,20,21,22].

To the authors’ knowledge, recent and thorough guidance supporting optimal prevention, recognition, and management of esophagitis and pneumonitis in NSCLC has not been published. We therefore convened a pan-Canadian multidisciplinary expert working group (EWG) to develop consensus recommendations and patient risk profiles to serve as practical resources for healthcare professionals (HCPs) treating patients with unresectable stage III NSCLC who are eligible for cCRT ± durvalumab.

## 2. Materials and Methods

### 2.1. Expert Working Group Selection

In early 2023, a multidisciplinary EWG was assembled that included nine HCPs: four radiation oncologists (A.M.B., H.B., A.B., D.S.), four medical oncologists (R.A.J., N.M., B.M., S.S.), and one nurse practitioner (C.L.). These individuals were chosen on the basis of their pan-Canadian representation, expertise in treatment of advanced NSCLC with cCRT ± durvalumab therapy, and experience managing esophagitis and pneumonitis. The inclusion of 9 experts was considered appropriate to assess consensus on clinical recommendations, as published literature recommends consultation with 5 to 12 individuals [33].

### 2.2. Preparation, Convergence, Consensus, and Reporting

**Figure 1:**
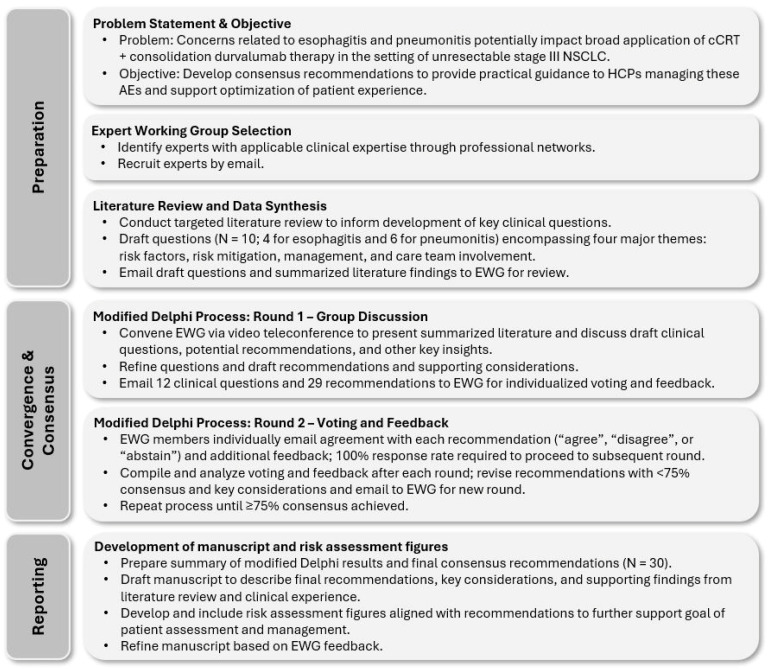
Summary of study methods. Additional details are provided in Supplementary Materials. AE, adverse event; cCRT, concurrent chemoradiation therapy; EWG, expert working group; HCPs, healthcare professionals; NSCLC, non-small-cell lung cancer.

A modified Delphi process was undertaken to develop clinical questions, recommendations, key considerations, and patient risk profiles relevant to esophagitis and pneumonitis arising during or after cCRT ± durvalumab therapy. Study methods are summarized in Figure 1, with additional details provided in Supplementary Materials.

## 3. Results and Discussion

**Figure 2:**
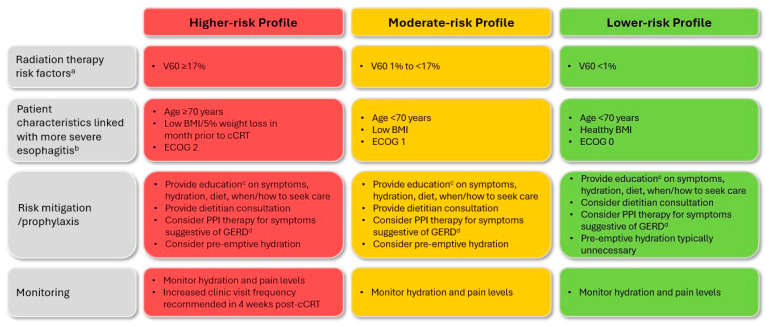
Patient risk profiles and associated guidance for cCRT-related esophagitis. Risk factors reflect those reported in published evidence and/or observed in real-world clinical practice—other considerations may apply. Patient risk should always be assessed on an individual basis. See full text for details. ^a^ Use of modern modulated planning techniques is recommended to mitigate esophagitis risk for all patients. ^b^ Other patient characteristics, such as female sex or the presence of GERD or dysphagia, may be relevant for high- or moderate-risk patients (mixed evidence). ^c^ Education should be provided to both patients and their caregivers/family, as appropriate. ^d^ Alternative agents (e.g., H2 receptor antagonists, sucralfate) may be preferred given risks associated with PPI therapy (see full text). Where possible, PPI therapy should be discontinued before initiation of durvalumab consolidation therapy. BMI, body mass index; cCRT, concurrent chemoradiation therapy; ECOG, Eastern Cooperative Oncology Group; GERD, gastroesophageal reflux disease; PPI, proton pump inhibitor; V60, esophagus volume receiving ≥60 Gy.

**Figure 3:**
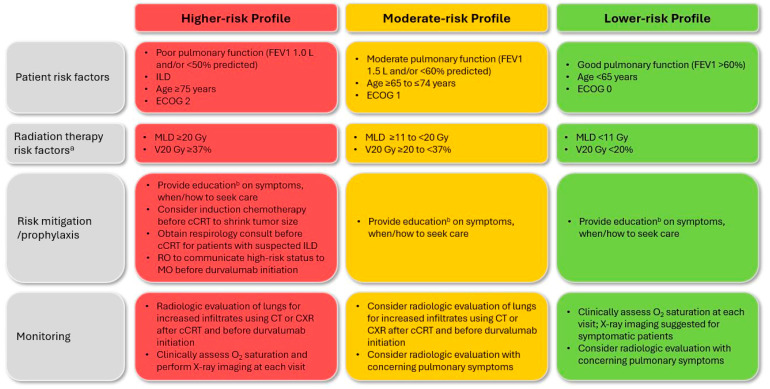
Patient risk profiles and associated guidance for pneumonitis related to cCRT ± consolidation durvalumab. Note: Risk factors reflect those reported in published evidence and/or observed in real-world clinical practice—other considerations may apply. Patient risk should always be assessed on an individual basis. See full text for details. ^a^ Use of modern modulated planning techniques is recommended to mitigate pneumonitis risk for all patients. ^b^ Education should be provided to both patients and their caregivers/family, as appropriate. cCRT, concurrent chemoradiation therapy; CT, computed tomography; CXR, chest X-ray; ECOG, Eastern Cooperative Oncology Group; FEV1, forced expiratory volume in 1 s; ILD, interstitial lung disease; MLD, mean lung dose; MO, medical oncologist; RO, radiation oncologist; V20, total lung volume ≥ 20 Gy.

Summaries of the final 12 clinical questions, 30 EWG recommendations, and key considerations for cCRT ± durvalumab-related esophagitis and pneumonitis are presented in Table 3 and Table 4, respectively, with expanded details and discussion of pertinent evidence, where available, provided below. A high level of consensus was achieved for all recommendations. Patient risk profiles and associated considerations are presented in Figure 2 (esophagitis) and Figure 3 (pneumonitis).

### 3.1. Esophagitis

**Question E1: How should patients be evaluated to ascertain the risk of developing esophagitis during or after cCRT?**
**Recommendation E1.1:** Before cCRT, all patients should be assessed for risk of developing esophagitis to inform a risk-adapted treatment approach. *(Level of agreement: 8 agree, 1 abstains)*
**Recommendation E1.2:** Radiation exposure to the esophagus is the most critical risk factor contributing to esophagitis during and/or after cCRT. *(Level of agreement: unanimous)*

As clinically relevant esophagitis is observed in approximately half of patients undergoing cCRT (Table 1), the risk of AE development and/or significant complications should be evaluated in advance of treatment initiation (Figure 2). Esophageal exposure to radiation therapy (RT) remains the primary risk factor [28]; as such, the EWG agreed that optimal RT selection, planning, and delivery is required to minimize the dose administered to the esophagus while maintaining target coverage (see Question E2) [26,34]. In a meta-analysis, higher esophagus volume receiving 60 Gy (V60; i.e., ≥17%) was associated with significantly increased esophagitis risk [17]. Acknowledging this, the EWG also agreed that longer esophagus length in field and larger mean esophageal and circumferential doses further increase risk.

In contrast to pneumonitis (see Question P1), baseline patient characteristics play a lesser role in esophagitis risk, but rather influence susceptibility to related complications. The EWG agreed that patients of advanced age (≥70 years) [35,36,37], with poor initial performance status [37] (i.e., Eastern Cooperative Oncology Group [ECOG] score of 2), low pre-treatment body mass index [38], or who have experienced 5% weight loss in the month before treatment [39] may be more likely to experience poor outcomes should esophagitis occur. The experts additionally recognized that female sex, gastroesophageal reflux disease (GERD), and baseline dysphagia may be associated with higher rates of severe acute esophagitis [28,37,40]. They indicated that such characteristics should be acknowledged before cCRT initiation, and that more frequent follow-up may be appropriate for these patients.

**Question E2: What measures should be undertaken to prevent or mitigate the risk of esophagitis during or after cCRT?**
**Recommendation E2.1:** Early education of patients and caregivers/family members is critical to provide information on potential signs and symptoms of esophagitis, hydration requirements, dietary management, and when to seek care. *(Level of agreement: unanimous)*
**Recommendation E2.2:** Appropriate planning techniques should be used to minimize exposure to radiation therapy. *(Level of agreement: unanimous)*
**Recommendation E2.3:** Dietitian consultation is recommended early in the course of cCRT for patients with high or moderate esophagitis risk. (Level of agreement: unanimous)
**Recommendation E2.4:** Proton pump inhibitor therapy should be considered for patients with symptoms suggestive of GERD. *(Level of agreement: unanimous)*

As in most disease settings, patient education on the potential side effects of cCRT in advanced NSCLC is critical not only to inform shared decision-making regarding initial treatment selection but also to encourage effective self-monitoring and timely and appropriate appeals for subsequent intervention. The EWG therefore emphasized that before initiation of cCRT, information on the characteristic signs and symptoms of esophagitis, typical timing of onset, and potential downstream sequelae should be discussed with both patients and caregivers/family members, as appropriate (Table 1). Patients should be directed to seek care at the first signs of dysphagia and/or odynophagia such that suitable medical intervention can be initiated (see Question E3). An elevated risk of dehydration, typically relating to inadequate fluid intake secondary to odynophagia, must be underscored and distinctive signs (e.g., dark urine coloration, dry mucus membranes, postural dizziness) communicated. The EWG noted that early intervention with pre-emptive outpatient hydration may be warranted for patients with high or moderate baseline risk: in a retrospective study, prophylactic intravenous (IV) hydration was associated with a significantly increased rate of cCRT completion and a significantly reduced incidence of acute grade ≥ 2 esophagitis [39].

As RT exposure is the main risk factor for esophagitis among patients receiving cCRT ± durvalumab, the EWG recommended selection of modern techniques enabling precise RT delivery. This includes prioritization of intensity-modulated RT (IMRT), volumetric modulated arc therapy (VMAT), or intensity-modulated proton therapy over two- or three-dimensional conformal RT, as well as avoidance of altered fractionation/dose-escalated delivery [16,28,37]. The experts additionally recommended limiting V60 (i.e., esophagus volume receiving ≥60 Gy) to <17% [17]. Furthermore, to ensure accurate reporting of RT planning metrics and in turn minimization of esophagitis risk, consistent contouring of the esophagus from the cricoid cartilage to the gastroesophageal junction was suggested. One expert emphasized that consideration of elective nodal RT should be judiciously weighed on a case-by-case basis against concerns regarding increased esophageal dose and risk of esophagitis.

Similarly to dehydration, malnutrition is a complication of lung cancer that not only arises from the cancer itself but also cCRT treatment and its associated AEs, including esophagitis. As noted in the joint European Society of Medical Oncology and European Society for Therapeutic Radiology and Oncology (ESMO-ESTRO) recommendations for cCRT supportive care, inadequate nutritional intake may result from treatment-induced esophagitis, dysphagia, anorexia, nausea, and psychosocial distress, among other factors [26]. Therefore, while acknowledging variations in geographic and center-related resources, the EWG recommended dietitian consultation for all patients, and particularly those with moderate or higher-risk profiles for esophagitis. The experts stated that initial consultation should occur before or early in the course of treatment, with regular follow-up undertaken during and/or after cCRT completion. Counseling should include adequate nutrition, including suitable food options and hydration requirements [26,29]. As per the ESMO/ESTRO recommendations, caloric intake should include a minimum of 30 kcal and 1.0 to 1.5 protein/kg of body weight in addition to recommended daily micronutrient allowances [26]. Food selections should be soft and non-irritating, with alcohol, coffee, and acidic and spicy options avoided [26,29]. The EWG noted that where feasible, culturally appropriate dietary information should be provided.

Given the potential for increased esophagitis risk, the EWG further recommended that all patients be monitored for symptoms suggestive of GERD, such as heartburn, regurgitation, and chest pain. Daily administration of a proton pump inhibitor (PPI) was stated to potentially be warranted [26]; however, as use of such therapy before and/or during IO may adversely affect the survival benefit of IO consolidation [41,42,43,44] and may also increase the risk of severe pneumonitis [43] and kidney-related AEs [45,46], alternative options such as histamine (H2) receptor antagonists or other coating agents (e.g., sucralfate) may be preferable. The experts underscored that if indicated, PPI therapy should be discontinued once esophagitis has fully resolved, and when possible, in advance of initiation of durvalumab consolidation therapy.

The EWG reviewed published literature for several other prophylactic options, including amifostine, indomethacin, naproxen, manuka honey, and glutamine [26,28]. Given lower levels of evidence and/or conflicting study findings, the group did not recommend use of these agents for prevention of esophagitis.

**Question E3: What treatments are effective for management of esophagitis?**
**Recommendation E3.1:** For symptomatic esophagitis, provide analgesics and consider dietitian support and PPI therapy if not already initiated; outpatient intravenous hydration can be initiated if patient is clinically dehydrated. Consider temporarily holding cCRT if initial measures prove ineffective. *(Level of agreement: unanimous).*
**Recommendation E3.2:** For esophagitis requiring hospitalization, consider holding or discontinuing cCRT (if ongoing), provide continuous intravenous hydration, optimize pain management, and increase dietitian support. *(Level of agreement: unanimous)*

Effective management of radiation-induced esophagitis varies on the basis of patient characteristics and AE grade (Table 2), but typically involves pain control, dietitian support, acid-blocking therapy, hydration, and/or holding cCRT [26,28,29]. As per the Common Terminology Criteria for Adverse Events (CTCAE) version 5.0 grading criteria [32], medical intervention is advised upon presentation of grade ≥ 2 events (Table 2). The EWG recommended that patients with symptomatic grade 2 esophagitis receive therapy for pain management, which may include analgesics (with or without viscous lidocaine) and over-the-counter antacid therapies. Prescription “magic mouthwash” formulations, which can include analgesic, steroid, antacid, antihistamine, antibiotic, and/or antifungal drugs, were flagged to be beneficial for symptom control (see Supplementary Materials for sample recipes). The experts stated that dietitian support should be increased or initiated if not already involved. Provision of PPI therapy was noted to reduce the time to heal erosive esophagitis [47] but should be discontinued after symptom resolution and ahead of IO initiation whenever feasible. The EWG recommended that patients experiencing clinical dehydration be considered for outpatient IV hydration at least two or three times weekly. As noted in the ESMO-ESTRO recommendations and by others, thrush and esophageal candidiasis frequently co-occur with esophagitis [26,28]; therefore, the experts suggested symptomatic or prophylactic treatment with antifungal agents (e.g., initially with nystatin; consider fluconazole for severe cases). Temporary hold of cCRT was recommended in the event that initial interventions fail to improve symptoms.

The EWG flagged that in severe cases of esophagitis in which eating and/or swallowing are significantly impaired (i.e., grade 3), hospitalization may be required to optimize pain management and administer continuous IV hydration. They noted that if cCRT persists, a hold or even permanent discontinuation should be considered. Dietary support should be increased; if oral nutritional deprivation has been or is anticipated to be prolonged and/or the patient experiences ≥5% loss of body weight despite maximal supportive care measures (as per ESMO-ESTRO) [26], enteral or parenteral feeding should be considered. The experts emphasized that when provided, enteral feeding via gastrostomy–jejunostomy (GJ) tube placement is preferred over parenteral support, given advantages related to safety, gut function, and cost [48]. For most cases, use of nasogastric tube feeding was not recommended, given the risk of symptom exacerbation and delayed recovery; however, it was recognized that some centers may be limited to or prefer this option.

**Question E4: Which HCPs should be involved in the care of patients who experience esophagitis?**
**Recommendation E4.1:** Multidisciplinary team care is essential for optimizing management of esophagitis and must involve shared responsibilities, clear communication, and collaboration. *(Level of agreement: 8 agree, 1 disagree)*
**Recommendation E4.2:** Patient follow-up frequency and HCP responsibility should be determined by esophagitis severity and timing of presentation. *(Level of agreement: unanimous)*
**Recommendation E4.3:** Healthcare professionals should share clear follow-up care instructions, including point-of-care contacts at the cancer center, during cCRT and consolidation treatment phases. *(Level of agreement: unanimous)*

Numerous publications have reported the importance of MDT collaboration in NSCLC [49], and the approach may be especially valuable in the setting of cCRT ± durvalumab consolidation therapy. The EWG emphasized that to optimize management, the MDT must comprise appropriate healthcare specialists who prioritize timely and effective communication tailored to patient characteristics, esophagitis risk factors, and the cCRT treatment experience. Team members will typically include radiation and medical oncologists, a dietitian, nurses, nurse practitioners, pharmacists, general practitioners in oncology, and potentially support from a geriatric oncologist. The experts stated that responsibility for follow-up care and primary communication should align with the stage of intervention and the identified risk of and/or timing of esophagitis presentation. They indicated that in most cases, the radiation oncologist should have primary responsibility for esophagitis monitoring, treatment, and follow-up, though cases with delayed occurrence or presenting in community settings may require management by other team members. The group agreed that follow-up frequency should be tailored to esophagitis risk or severity—weekly visits with an appropriate MDT member were recommended for patients with ongoing symptoms after cCRT. The EWG emphasized that the gap phase between end of cCRT and start of durvalumab consolidation (typically about four weeks) can be an especially vulnerable time for patients that requires diligent monitoring. This relates to a peak in the occurrence of delayed-onset toxicities (including esophagitis) and the reduced frequency of clinic visits during transfer of care. The experts underscored that where applicable, MDT communications should highlight factors that may influence the treatment approach for IO consolidation therapy, such as the presence of comorbidities or elevated risk related to the RT plan (e.g., significant esophagus coverage; see Question E1). Patients and their caregivers/family members, as appropriate, must be provided clear follow-up instructions, including specific point-of-care contacts in the event of AE onset or worsening.

### 3.2. Pneumonitis

**Question P1: How should patients be evaluated to ascertain the risk of developing pneumonitis after cCRT ± durvalumab consolidation?**
**Recommendation P1.1:** Before initiating cCRT ± durvalumab, all patients should be assessed for risk of developing pneumonitis to inform a risk-adapted treatment approach. *(Level of agreement: unanimous)*
**Recommendation P1.2:** Key factors identified to increase risk of symptomatic pneumonitis include large radiation volume (V20, mean lung dose) and poor lung function or the presence of interstitial lung disease at baseline. *(Level of agreement: unanimous)*

Pneumonitis is one of the most severe and potentially life-threatening treatment-related AEs associated with cCRT ± durvalumab consolidation therapy (Table 1) [30]. As such, assessment of baseline risk ahead of treatment initiation is particularly important, given lung function may already be compromised because of disease characteristics (e.g., tumor size, location) and/or lung comorbidities [30].

Similarly to esophagitis, a primary risk factor for radiation pneumonitis (RP) is the RT treatment plan. Both older RT techniques and larger radiation volumes are associated with a significantly increased incidence of symptomatic (grade ≥ 2) RP (Figure 3) [18,19,26,27,50]. Specifically, lung V20 (i.e., total lung dose ≥ 20 Gy) and mean lung dose (MLD; the mean dose to total lung volume minus planning target volume) are well correlated with RP risk [4,18,19,23,26]. Another significant predictor is the amount of RT delivered to the heart [51]. Although not RT-related, concurrent chemotherapy type can also impact RP risk [27,50]. Notably, although evidence is somewhat mixed, the risk of RP or IO-related pneumonitis does not appear to be impacted by the interval between cCRT completion and initiation of durvalumab consolidation therapy [25,52,53].

In contrast to esophagitis, patient- and disease-related characteristics may play a more predominant role in the risk of pneumonitis after cCRT ± durvalumab. The presence of pre-existing interstitial lung disease (ILD; including subclinical) is associated with a particularly elevated risk of both RP and IO-related events [54,55,56,57,58,59]. Additionally, as reported in a subanalysis of the PACIFIC trial [60] and by others [61,62], patients of Asian descent have a higher risk of RP and IO-related pneumonitis. Multiple other factors—age; sex; World Health Organization (WHO) performance status (PS); smoking status; tumor histology, size, and location; history of chronic obstructive pulmonary disease or asthma; diffusing capacity of the lung for carbon monoxide; and prior lung surgery [16,27,29,50,63,64]—have been linked, at least in some studies, with an elevated risk of RP or higher-grade RP.

**Question P2: What measures should be undertaken to prevent or mitigate the risk of pneumonitis after cCRT ± durvalumab consolidation?**
**Recommendation P2.1:** Strategies should be implemented to reduce the volume of radiation delivered to normal structures and to address other modifiable risk factors. *(Level of agreement: 8 agree, 1 abstains)*
**Recommendation P2.2:** For patients at very high risk of pneumonitis, determine whether definitive cCRT ± durvalumab consolidation is appropriate and safe to deliver on a case-by-case basis. *(Level of agreement: unanimous)*

As several of the risk factors for pneumonitis are unmodifiable patient- and/or disease-related characteristics (see Question P1), mitigation approaches primarily include appropriate patient selection via baseline risk assessment and minimization of RT exposure (Figure 3). Regardless of risk level, the EWG recommended that all patients undergoing cCRT ± durvalumab consolidation, as well as their caregivers/family members, as appropriate, receive counseling regarding the signs of initial or worsening pneumonitis, as well as point-of-care contact information. As for esophagitis, prioritization of newer and more targeted RT techniques such as IMRT and VMAT was encouraged [65]. Furthermore, in alignment with other guidelines [8,26,34], the experts agreed that V20 and MLD should be kept below 37% and 20 Gy, respectively, to minimize RP risk. The importance of limiting heart dose was also recognized: in a study by Gao and colleagues, mean heart dose ≥ 5 Gy was a significant predictor of pneumonitis risk [51]. Use of respiratory motion management and treatment verification strategies, as described by the European Organisation for Research and Treatment of Cancer and National Comprehensive Cancer Network (NCCN) [5,34], were additionally recommended.

For patients at especially high risk of pneumonitis (Figure 3), the EWG recommended individualized consideration of the appropriateness of cCRT ± durvalumab. The importance of direct communication between the radiation and medical oncologist was underscored, particularly to inform the decision to initiate consolidation therapy (see Question P8). The experts suggested that alternative treatment strategies may be suitable for higher-risk unresectable patients, such as use of induction chemotherapy to potentially shrink tumor size or RT planning to meet dose constraints. As per the Society for Immunotherapy of Cancer (SITC) guidelines [63], the experts agreed that patients with suspected autoimmune ILD should be referred to a specialist before initiation of cCRT ± durvalumab such that pulmonary function tests and other risk assessments can be conducted. The group additionally acknowledged that although contradictory findings have been published, certain chemotherapeutic agents may pose an increased risk of pneumonitis (e.g., taxane-based therapies) [16,27,29,50], and thus selection should be determined appropriately.

Using results from the targeted literature review, the EWG also considered evidence for numerous investigational approaches to mitigating radiation-induced lung toxicity. Therapies included amifostine, angiotensin-converting enzyme inhibitors (ACEIs) and angiotensin II receptor subtype 1 (AT-1) antagonists, pentoxifylline, pirfenidone, and pamrevlumab, among others [27]. Although data for ACEIs and AT-1 antagonist appeared promising, the group concluded that more robust evidence is needed for all options to fully understand their clinical benefit.

**Question P3: What approach is recommended to determine the etiology of symptomatic pneumonitis?**
**Recommendation: P3.1:** Presentation of pneumonitis during durvalumab consolidation therapy may reflect RP or IO-related pneumonitis. Patients should be assessed by the treating radiation oncologist to help determine underlying etiology. *(Level of agreement: 8 agree, 1 disagree)*
**Recommendation P3.2:** The radiation oncologist should compare the radiation plan with changes on CT imaging. In RP, lung parenchymal changes generally conform to the radiation treatment field, while IO-related pneumonitis is more likely to present with bilateral or diffuse lung changes. *(Level of agreement: unanimous)*

Per the PACIFIC trial, initiation of durvalumab consolidation therapy is recommended to occur between 1 and 42 days of successful cCRT completion [2,8]; however, onset of symptomatic RP typically occurs between one and six months after cCRT [26,27]. Therefore, as RP frequently presents after IO administration has already occurred, it is important to differentiate RP and IO-related pneumonitis to guide appropriate management approaches (see Questions P4 and P5) and define responsible care team members (see Question P8).

Although RP can develop one to six months after cCRT, onset usually occurs within three months of completion (Table 1) [27]. Visualized using chest CT imaging, the AE commonly presents within a sharp border confined to the irradiated treatment field (typically within the 20 Gy isodose line) and may include ground-glass opacities with or without airspace consolidation [27,31,66,67]. Ipsilateral pleural effusion may develop concurrently or alongside lung atelectasis [66]. In contrast, IO-related pneumonitis has a more variable time to onset: a median of 2.6 months from IO initiation has been reported, with a range spanning <0.5 to 19.2 months [30]. The IO-induced presentation is more likely to be characterized by bilateral or diffuse lung changes [31], with pulmonary opacities observed outside the involved RT field [20].

Considering these characterizations, as well as the potential for inaccurate diagnosis of IO-related pneumonitis and inappropriate discontinuation of durvalumab therapy [68], the EWG recommended that the treating radiation oncologist compare the RT plan with changes observed via CT to determine pneumonitis etiology. One expert stressed that for some patients, etiology is especially difficult to distinguish and both cCRT and durvalumab may contribute to AE presentation. Multidisciplinary team discussion may therefore be required for definitive diagnosis of pneumonitis origin (see Question P8). Increased vigilance was recommended for presentations associated with such uncertainty, with a management approach aligned to that recommended for IO-related cases (see Question P5). The EWG acknowledged that CT radiomics and machine learning have recently shown success in distinguishing between pneumonitis types [31,69,70], and although not routinely integrated into clinical practice, these techniques may soon regularly support identification of pneumonitis etiology and clinical decision-making.

**Question P4: What treatments are effective for management of RP?**
**Recommendation P4.1:** Asymptomatic (grade 1) RP is common after cCRT and does not warrant investigation or treatment; however, increased monitoring may be warranted for patients presenting with new radiological changes after initiation of durvalumab consolidation therapy. *(Level of agreement: unanimous)*
**Recommendation P4.2:** Patients with confirmed grade 2 RP should be followed under close observation. Prompt initiation of corticosteroid therapy should be considered in the event of worsening symptoms, as well as supplemental oxygen as clinically appropriate. Consider holding durvalumab if patient has initiated consolidation therapy. *(Level of agreement: unanimous)*
**Recommendation P4.3:** Patients with confirmed grade 3/4 RP should promptly receive corticosteroid therapy and supplemental oxygen as clinically appropriate. Consider whether referral to respirology and/or hospitalization are warranted. Hold durvalumab if patient has initiated consolidation therapy. *(Level of agreement: unanimous)*
**Recommendation P4.4:** Recommended corticosteroid therapy is oral prednisone 1 mg/kg/day up to 60 mg. Treatment should be tapered slowly over a duration of at least 6 weeks once RP has clinically improved. Simultaneous initiation of PPI therapy is also recommended, as well as consideration of prophylaxis for PJP. *(Level of agreement: unanimous)*

In agreement with formal guidelines and recommendations from other expert groups [26,30,50,63,71,72,73], the EWG stated that management of pneumonitis should correlate with grade of severity. Additionally, although treatment protocols are relatively similar, approaches should be tailored to the etiology of the AE (see Question P3): for cases in which origin is unclear, IO-related protocols should be followed (see Question P5). The EWG agreed that although asymptomatic grade 1 RP occurs frequently after cCRT, this presentation does not typically require further investigation or treatment; however, observation of novel radiological changes after durvalumab consolidation should prompt increased clinical monitoring. For patients with symptomatic grade ≥ 2 RP, the experts concurred that corticosteroid therapy remains the mainstay of treatment [26,27]. They stated that although grade 2 RP can initially be followed under close observation (e.g., every one to two weeks), changes on chest X-ray and/or symptom worsening necessitate initiation of corticosteroid therapy and potentially supplemental oxygen. If already initiated, a hold of durvalumab consolidation therapy should be considered until corticosteroid therapy (or equivalent) is tapered to ≤10 mg/day. For patients with grade 3 or 4 RP, the experts recommended immediate treatment with corticosteroid therapy, provision of supplemental oxygen, and hold of durvalumab if initiated. Respirology referral and potentially hospitalization were encouraged for patients requiring oxygen supplementation.

The EWG stated that initial corticosteroid therapy should include oral prednisone 1 mg/kg body weight per day, up to a total of 60 mg per day (or equivalent); dexamethasone may be considered for patients not requiring rapid symptom control. The experts agreed that once RP has clinically improved, corticosteroid therapy should be tapered over a duration of six weeks or even longer for some individuals—abrupt discontinuation must be avoided to prevent AE relapse [27]. Given the potentially deleterious effects of corticosteroids on bone, the EWG suggested consideration of treatment with calcium, vitamin D, and/or bisphosphonates. Prophylaxis for pneumocystis jiroveci pneumonia (PJP) was also recommended, along with careful initiation of PPI therapy and management of comorbid conditions (e.g., those potentially worsened by steroid therapy, such as diabetes). As flagged for esophagitis, the EWG underscored that where possible, PPI duration should be limited to the corticosteroid treatment phase to mitigate a potentially negative interaction with IO. Criteria for initiating durvalumab treatment and restarting the IO were also discussed (see Questions P6 and P7, respectively).

**Question P5: What treatments are effective for management of IO-related pneumonitis?**
**Recommendation P5.1:** Patients with asymptomatic (grade 1) IO-related pneumonitis should receive more frequent follow-up with oxygen saturation and chest X-ray. Consider holding durvalumab on a case-by-case basis. *(Level of agreement: 8 agree, 1 abstains)*
**Recommendation P5.2:** Patients with confirmed grade 2 IO-related pneumonitis should have durvalumab suspended, corticosteroid therapy promptly initiated, and supplemental oxygen provided as clinically appropriate. Monitor closely; if pneumonitis persists or worsens after 48 to 72 h, treat as grade 3 or 4. *(Level of agreement: 7 agree, 2 abstain)*
**Recommendation P5.3:** Patients with confirmed grade 3/4 IO-related pneumonitis should have durvalumab suspended or discontinued and corticosteroid therapy promptly initiated. Supplemental oxygen, hospitalization, and respirology referral are warranted. If pneumonitis persists or worsens after 48 h, consider initiating a non-steroidal immunosuppressive agent. *(Level of agreement: 8 agree, 1 abstains)*
**Recommendation P5.4:** Recommended outpatient corticosteroid therapy is oral prednisone 1–2 mg/kg/day; treatment should be tapered slowly over a duration of at least 6 weeks once pneumonitis has clinically improved. Simultaneous initiation of PPI therapy is recommended, as well as consideration of prophylaxis for PJP. *(Level of agreement: 8 agree, 1 abstains)*

Detailed protocols for management of IO-related pneumonitis are available from several groups [63,71,72,73] and were considered by the EWG. As highlighted in Questions P3 and P4, the experts stressed the importance of determining pneumonitis etiology to inform treatment and that cases of uncertain origin should be managed as per an IO-related pneumonitis protocol.

For patients determined to have asymptomatic grade 1 IO-related pneumonitis, the EWG recommended increased follow-up to monitor oxygen saturation and changes on chest X-ray. The group indicated that although the decision to continue durvalumab consolidation should be individualized, therapy would typically be held for patients with ILD who develop superimposed IO-related pneumonitis. For patients with grade 2 pneumonitis, the experts recommended that durvalumab be held and corticosteroids initiated; if symptoms do not improve within 48 to 72 h of such therapy, the AE should be treated as grade 3/4. The EWG stated that for grade 3/4 pneumonitis, hospitalization and respirology referral should be considered in addition to durvalumab hold and initiation of corticosteroid therapy. The group noted that escalation of corticosteroids and/or additional immunosuppression with non-steroidal options should be considered for patients who do not show improvement within 48 h.

In terms of specific drug therapy, the EWG recommended oral prednisone 1 to 2 mg/kg/day; for patients admitted to hospital, IV methylprednisolone (2–4 mg/kg/day) may be preferable [72,73]. Use of dexamethasone was not recommended, given its potentially delayed onset of action. As for RP, the experts stated that once IO-related pneumonitis has clinically improved, corticosteroid therapy should be tapered over a duration of at least six weeks to avoid relapse. For patients without improvement, the EWG recommended administration of IV infliximab (5 mg/kg) or mycophenolate mofetil (1–1.5 g twice daily); infliximab may be repeated after 14 days if no improvement is observed. As for RP, prophylaxis with calcium, vitamin D, and/or oral bisphosphonates was recommended to decrease the effects of corticosteroids on bone. Moreover, simultaneous initiation of PPI therapy (with discontinuation before durvalumab re-initiation) and consideration of PJP prophylaxis were also suggested. For all patients, the experts noted that referral to an infectious disease specialist may be warranted if viral or bacterial infection (e.g., pneumococcus, legionella) is suspected; empiric use of broad-spectrum antibiotics may be justified in some cases. Given the risk of recurrent pneumonitis after symptom improvement, both in patients who undergo durvalumab re-initiation (see Question P7) and those who do not, the EWG additionally recommended careful ongoing and intensified monitoring of all patients.

**Question P6: Which patients with RP are suitable for initiation of durvalumab consolidation?**
**Recommendation P6.1:** Consider initiating durvalumab for patients with asymptomatic RP after cCRT; more frequent follow-up with chest X-ray may be warranted. *(Level of agreement: unanimous)*

Most patients who undergo cCRT experience asymptomatic lung changes suggestive of grade 1 RP. Furthermore, only a limited proportion experience symptomatic RP before durvalumab consolidation is initiated, as the average time between cCRT completion and durvalumab initiation is typically less than two months. The EWG underscored that initiation of durvalumab is inadvisable for patients with ongoing symptoms; consolidation IO should only be considered for those who have become asymptomatic (grade ≤ 1). Follow-up should include chest X-ray at a frequency corresponding to the patient’s risk of deterioration and/or hospitalization. Higher-risk cases, such as patients who experience more severe grades of RP or who have comorbidities, should undergo monthly or even weekly or biweekly follow-up and imaging.

**Question P7: Which patients are suitable for durvalumab re-initiation after resolution of pneumonitis?**
**Recommendation P7.1:** For patients with confirmed RP who have resolution of symptoms and corticosteroids tapered to ≤10 mg/day, consider re-initiating durvalumab. *(Level of agreement: unanimous)*
**Recommendation P7.2:** For patients with IO-related pneumonitis who have symptom resolution and corticosteroids tapered to ≤10 mg/day, the decision to re-initiate durvalumab should be individualized on the basis of patient characteristics and shared decision-making. *(Level of agreement: 8 agree, 1 disagree)*

Given the proven efficacy of durvalumab consolidation therapy in unresectable stage III NSCLC [2,3], as well as evidence suggesting that early termination of such treatment is associated with significantly reduced survival [74], it has been of interest to understand whether re-initiation of durvalumab after a pneumonitis-related hold is safe for patients. Although studies remain limited in this setting, available evidence suggests that therapy can and should be re-initiated for some patients, especially those who have received less than two months of consolidation therapy [19,23,74,75,76]. One of the largest evaluations of this topic was the HOPE-005/CRIMSON study, which retrospectively examined the impact of pneumonitis on durvalumab use among patients with NSCLC receiving cCRT [19]. In total, 52 of 225 patients (23%) who received durvalumab were treated with corticosteroids for pneumonitis. Of these patients, 21 (40%) were rechallenged with the IO therapy, of whom 6 (29%) experienced pneumonitis relapse (all grade 2). Notably, at the time of rechallenge, first pneumonitis had not resolved to grade ≤ 1 or steroid dosing was not tapered to ≤10 mg/day in two of the six relapsed patients. Three of the six patients ultimately discontinued durvalumab because of pneumonitis relapse. The findings of HOPE-005/CRIMSON align with those from a large study of immune-related adverse events (irAEs) captured in the World Health Organization’s Vigibase database that considered multiple disease settings and IO therapies (N = 6123 with IO re-initiation) [77]. In this study, 28.8% of patients experienced a recurrence of the same irAE after re-initiation of the same IO therapy; in multivariate analysis, the odds of recurrent pneumonitis were significantly increased after resumption of IO. Interestingly, the study also reported that the initial occurrence of immune-related pneumonitis occurred substantially later among patients who experienced a recurrence of the irAE (88 vs. 44 days for non-recurring patients).

Considering the evidence, re-initiation of durvalumab consolidation therapy appears to be feasible and generally well tolerated for some patients. Nonetheless, as per guideline recommendations from SITC [63] and NCCN (v. 1.2024) [71], the EWG emphasized that the decision to restart the IO must be individualized on the basis of pneumonitis severity and status, as well as patient characteristics and needs. As stated by SITC, clinicians must consider whether the initial irAE was severe or life-threatening and understand whether the patient requires prolonged or multiple immunosuppressant therapies for resolution [63]. The EWG additionally recommended consideration of patient age and level of PD-L1 expression [3], as well as issues relevant to shared decision-making—patients’ AE experience, personal preferences, treatment goals, and proximity to the cancer center should all be discussed to inform the optimal path forward.

In general, similarly to the NCCN guidelines for management of IO-related toxicities [71], the EWG suggested that patients with confirmed grade 1 or grade 2 pneumonitis who experience symptom resolution and achieve corticosteroid tapering to ≤10 mg/day can typically be considered for durvalumab re-initiation. Radiographic evidence of improvement may further support restarting IO. However, the group recommended increased caution when re-initiating durvalumab among individuals who experience grade 3 pneumonitis. The EWG indicated that among such patients, careful retrial may be warranted for those who demonstrate a prompt and complete response to corticosteroid therapy. Irrespective of etiology, durvalumab re-initiation was not recommended for patients who experience grade 4 pneumonitis events. As per NCCN recommendations [71], the experts agreed that if durvalumab is restarted and symptomatic pneumonitis returns, the IO should be permanently discontinued.

**Question P8. Which HCPs should be involved in the care of patients who experience pneumonitis?**
**Recommendation P8.1:** Multidisciplinary team care is essential for optimizing follow-up of pneumonitis and must involve shared responsibilities, clear communication, and collaboration. *(Level of agreement: 8 agree, 1 disagree)*
**Recommendation P8.2:** Radiation and medical oncologists should communicate regarding patient status and classification of pneumonitis etiology. If RP, the radiation oncologist should be responsible for management and follow-up until resolution; if IO-related, the medical oncologist should be responsible. *(Level of agreement: unanimous)*

As stated for esophagitis, MDT involvement is critical for optimal care of NSCLC and may be particularly relevant for diagnosis and management of pneumonitis after cCRT ± durvalumab. The importance of this approach has already been emphasized in the literature: in the HOPE-005/CRIMSON study, the authors concluded that medical and radiation oncologist cooperation is needed to ensure safe administration of cCRT and completion of durvalumab consolidation therapy [19]. Moreover, in a retrospective, single-center study of patients receiving the PACIFIC regimen, multidisciplinary review of CT scans, RT doses, and patient symptoms was found to increase the accuracy of diagnosing pneumonitis etiology and help guide care [68]. The EWG therefore recommended that ongoing collaboration and effective communication occur between radiation and medical oncologists. Timely and clear discourse regarding patient comorbidities and pneumonitis etiology was flagged to be especially pertinent, given their impact on AE risk and treatment protocols. The experts advised that where feasible, pneumonitis origin should dictate responsibility for management and follow-up: the radiation oncologist should lead the care of patients presenting with RP, while the medical oncologist should lead care of IO-related cases. In the event of uncertain etiology, care responsibilities should be shared. As appropriate, inclusion of a nurse, nurse practitioner, and the primary care practitioner was encouraged, as well as involvement of respirology, radiology, and/or infectious disease specialists as needed.

## 4. Conclusions

As the current SoC for unresectable stage III NSCLC, cCRT + durvalumab should be considered for all eligible patients. Optimized risk assessment and management of radiation esophagitis is recommended to minimize impact on patient health and QoL, in addition to facilitating timely initiation of durvalumab consolidation after completion of cCRT. Similarly, although RP and IO-related pneumonitis can limit administration of durvalumab, opportunities exist for risk mitigation and effective management that can minimize or even eliminate patient-, HCP-, and system-related burdens. These consensus guidelines support increased HCP comfort with ascertaining patient risk and addressing these AEs should they arise among patients with unresectable stage III NSCLC. They also encourage efficient intercommunication among MDT members that will ideally support a maximized patient treatment experience and outcome. Finally, these recommendations are expected to offer utility in other patient populations treated with cCRT ± immunotherapy.

## Figures and Tables

**Table 1:** Characteristics of cCRT ± durvalumab-induced esophagitis and pneumonitis.

	Esophagitis	Pneumonitis
Frequency	Symptomatic grade ≥ 2: ~50% [17]	Grade ≥ 2 RP: 30–44% [18,23,24] Any-grade IO-related: 11–22% ^a^ (grade 3/4: 1.9%) [20,25]
Typical time to onset (any grade)	2–3 weeks after initiation of cCRT [26] ^b^	RP: 1–6 months after cCRT [26,27] IO-related: 54 days (median) after durvalumab initiation (range, 2–342 days) [25]
Typical time to resolution	~8 weeks after end of cCRT [26]	53 days (median; range, 22–588 days) [25]
Signs and symptoms [16,26,28] ^c^	Dysphagia Odynophagia Acid reflux and heartburn Substernal discomfort Anorexia	Dyspnea during rest or exertion Cough Fatigue Hypoxia or decrease in SpO_2_ from baseline Low-grade fever Chest discomfort Pleuritic pain
Risks and complications [16,26,27,28,29]	Dehydration ^d^ Electrolyte imbalance Weight loss Malnutrition Impaired QoL Aspiration Treatment interruption or discontinuation Hospitalization Enteral or parenteral nutrition (rare) Esophageal stricture (rare) Esophageal perforation (rare) Bleeding (rare)	Treatment interruption or discontinuation Corticosteroid- or other immunosuppressant-related AEs Impaired QoL Oxygen dependence Weight loss Hospitalization Permanent pulmonary fibrosis Mortality (rare)
Differentiation of etiology [30,31] ^e^	N/A	RP: Changes typically conform to radiation treatment field with sharp border. IO-related pneumonitis: Typically, bilateral or diffuse lung changes.

^a^ Symptomatic (grade ≥ 2) IO-related events were not reported separately. ^b^ Presentation of esophagitis is typically acute; however, late presentation (>3 months after completion of cCRT) has been described (median onset: 6 months). ^c^ Patients with grade 1 esophagitis and/or pneumonitis are asymptomatic; see Table 2. ^d^ Dehydration may be characterized by dark-colored urine, dry mucus membranes, and/or postural dizziness. ^e^ Differentiation based on CT imaging. AE, adverse event; cCRT, concurrent chemoradiation therapy; CT, computed tomography; IO, immuno-oncology therapy; N/A, not applicable; QoL, quality of life; RP, radiation pneumonitis; SpO_2_, oxygen saturation.

**Table 2:** Grading of esophagitis and pneumonitis (CTCAE version 5.0 [32]).

Grade	Esophagitis ^a^	Pneumonitis
1	Asymptomatic Clinical or diagnostic observations only—intervention not indicated.	Asymptomatic Clinical or diagnostic observations only—intervention not indicated.
2	Symptomatic; altered eating or swallowing Oral supplements indicated	Symptomatic; limiting instrumental ADL Medical intervention indicated
3	Severely altered eating/swallowing Tube feeding, TPN, or hospitalization indicated	Severe symptoms; limiting self-care ADL Oxygen indicated
4	Life-threatening consequences Urgent operative intervention indicated	Life-threatening respiratory compromise Urgent intervention indicated (e.g., tracheotomy or intubation)
5	Death	

^a^ Dysphagia, a symptom of esophagitis, may also be assessed and graded using CTCAE v.5.0 criteria. Grade 1 dysphagia is symptomatic, though patients can eat a regular diet. Other grades of dysphagia are similar to those for grades 2 to 5 esophagitis. ADL, activities of daily living; CTCAE, Common Terminology Criteria for Adverse Events; RP, radiation pneumonitis; TPN, total parenteral nutrition.

**Table 3:** Esophagitis: summary of clinical questions, recommendations, and key considerations.

**Target Population**	Patients with stage III unresectable NSCLC treated with cCRT ± durvalumab consolidation therapy	
**Target Audience**	Radiation oncologists; medical oncologists; oncology nurses, nurse practitioners, and physician assistants; respirologists; pulmonologists; radiologists; family physician/community primary care team members; geriatric oncologists; pharmacists; patients	
**Clinical Question**	**Recommendations and Level of Agreement**	**Key Considerations**
E1. How should patients be evaluated to ascertain the risk of developing esophagitis during or after cCRT?	**Recommendation E1.1:** Before cCRT, all patients should be assessed for risk of developing esophagitis to inform a risk-adapted treatment approach. *(Level of agreement: 8 agree, 1 abstains)* **Recommendation E1.2:** Radiation exposure to the esophagus is the most critical risk factor contributing to esophagitis during and/or after cCRT. *(Level of agreement: unanimous)*	*See risk profile considerations in Figure 2.* Patients presenting with higher frailty/ECOG 2, advanced age, and/or low pre-treatment BMI may be more susceptible to adverse outcomes related to esophagitis. Key RT parameters impacting risk of esophagitis include volume of esophagus receiving 60 Gy (≥17%), length of esophagus in field, mean esophageal dose, and circumferential dose.
E2. What measures should be undertaken to prevent or mitigate the risk of esophagitis during or after cCRT?	**Recommendation E2.1:** Early education of patients and caregivers/family members is critical to provide information on potential signs and symptoms of esophagitis, hydration requirements, dietary management, and when to seek care. *(Level of agreement: unanimous)* **Recommendation E2.2:** Appropriate planning techniques should be used to minimize exposure to RT. *(Level of agreement: unanimous)* **Recommendation E2.3:** Dietitian consultation is recommended early in the course of cCRT for patients with high or moderate esophagitis risk. *(Level of agreement: unanimous)* **Recommendation E2.4:** Proton pump inhibitor therapy should be considered for patients with symptoms suggestive of GERD. *(Level of agreement: unanimous)*	Counseling must be provided on potential signs/symptoms and timing of appearance, characteristics of dehydration, and adequate maintenance of fluid and caloric intake. Patients should be instructed to seek clinical care at the first signs of dysphagia or odynophagia; caregivers/family members should also be educated on triggers for seeking care. Modern RT approaches (e.g., VMAT/IMRT) should be prioritized where feasible, with esophagus V60 <17%. Consistent esophagus contouring (cricoid to the GEJ) is important for accurate reporting of RT planning metrics and assessment of esophagitis risk. The need for elective nodal RT should be carefully evaluated in relation to patient risk/benefit. Pre-emptive dietitian consultation may be considered for all patients; however, it is recognized that resources may be limited. At a minimum, patients with high or moderate risk should be considered. Early intervention with pre-emptive hydration may be appropriate for patients with high or moderate risk. If indicated, PPI therapy should continue until esophagitis symptoms have resolved; therapy should be discontinued upon symptom resolution and before initiation of IO consolidation therapy whenever possible.
E3. What treatments are effective for management of esophagitis?	**Recommendation E3.1:** For symptomatic esophagitis, provide analgesics and consider dietitian support and PPI therapy if not already initiated; outpatient IV hydration can be initiated if patient is clinically dehydrated. Consider temporarily holding cCRT if initial measures prove ineffective. *(Level of agreement: unanimous)* **Recommendation E3.2:** For esophagitis requiring hospitalization, consider holding or discontinuing cCRT (if ongoing), provide continuous IV hydration, optimize pain management, and increase dietitian support. *(Level of agreement: unanimous)*	Varying formulations of “magic mouthwash” may be available that can provide effective combination therapy (see Supplementary Materials). If PPI therapy is indicated, treatment should continue until esophagitis symptoms have resolved and be discontinued before initiation of IO consolidation therapy whenever possible. Consider symptomatic or prophylactic treatment with antifungals given frequent co-occurrence of thrush and candidiasis. GJ tube placement should only be considered for severe cases in which nutritional deprivation is anticipated to be prolonged. Given superior ease of use and reduced resource requirements, enteral nutritional support is preferred over total parenteral.
E4. Which HCPs should be involved in the care of patients who experience esophagitis?	**Recommendation E4.1:** Multidisciplinary team care is essential for optimizing management of esophagitis and must involve shared responsibilities, clear communication, and collaboration. *(Level of agreement: 8 agree, 1 disagree)* **Recommendation E4.2:** Patient follow-up frequency and HCP responsibility should be determined by esophagitis severity and timing of presentation. *(Level of agreement: unanimous)* **Recommendation E4.3:** Healthcare providers should share clear follow-up care instructions, including point-of-care contact(s) at the cancer center, during cCRT and consolidation treatment phases. *(Level of agreement: unanimous)*	MDT may include the RO, MO, dietitian, nurse, NP, and pharmacist; consider referral to geriatric oncologist for frail, elderly patients. Patients may be most vulnerable in the first four weeks after cCRT given transition between cCRT and consolidation IO therapy; provision of clear care instructions and contact information to patients and caregivers/family members is particularly important during this phase. More frequent follow-up visits (e.g., weekly) are recommended for patients with ongoing symptomatic esophagitis. PCP should receive ongoing documentation regarding patient management approach and status.

Abbreviations: BMI, body mass index; cCRT, concurrent chemoradiation therapy; ECOG 2, Eastern Cooperative Oncology Group 2; GEJ, gastroesophageal junction; GERD, gastroesophageal reflux disease; GJ, gastrojejunostomy; HCP, healthcare professional; IMRT, intensity-modulated radiation therapy; IO, immuno-oncology therapy; IV, intravenous, MDT, multidisciplinary team; MO, medical oncologist; NP, nurse practitioner; PCP, primary care practitioner; PPI, proton pump inhibitor; RO, radiation oncologist; RT, radiation therapy; TPN, total parenteral nutrition; VMAT, volumetric modulated arc therapy.

**Table 4:** Pneumonitis: summary of clinical questions, recommendations, and key considerations.

**Target Population**	Patients with stage III unresectable NSCLC treated with cCRT ± durvalumab consolidation therapy	
**Target Audience**	Radiation oncologists; medical oncologists; oncology nurses, nurse practitioners, and physician assistants; respirologists; pulmonologists; radiologists; family physician/community primary care team members; geriatric oncologists; pharmacists; patients	
**Clinical Question**	**Recommendation and Level of Agreement**	**Key Considerations**
P1. How should patients be evaluated to ascertain the risk of developing pneumonitis after cCRT ± durvalumab consolidation?	**Recommendation P1.1:** Before initiating cCRT ± durvalumab, all patients should be assessed for risk of developing pneumonitis to inform a risk-adapted treatment approach. *(Level of agreement: unanimous)* **Recommendation P1.2:** Key factors identified to increase the risk of symptomatic pneumonitis include large radiation volume (V20, MLD) and poor lung function or presence of ILD at baseline. *(Level of agreement: unanimous)*	*See risk profile considerations in Figure 3.* Larger radiation volumes (lung V20 ^a^ ≥ 37%, MLD ≥ 20 Gy; typically driven by tumor size, location, and/or extent) and poorer baseline lung function are significant risk factors for RP. ILD is a significant risk factor for high-grade/fatal RP and/or IO-related pneumonitis yet may go unrecognized; consider radiology review or respirology/pulmonology consultation at baseline to rule out subclinical ILD if CT findings are equivocal. Other factors, such as advanced patient age and Asian ethnicity, may have a more modest impact on risk but should be considered.
P2. What measures should be undertaken to prevent or mitigate the risk of pneumonitis after cCRT ± durvalumab consolidation?	**Recommendation P2.1:** Strategies should be implemented to reduce the volume of radiation delivered to normal structures and to address other modifiable risk factors. *(Level of agreement: 8 agree, 1 abstains)* **Recommendation P2.2:** For patients at very high risk of pneumonitis, determine whether definitive cCRT ± durvalumab consolidation is appropriate and safe to deliver on a case-by-case basis. *(Level of agreement: unanimous)*	Patients and caregivers/family members, as appropriate, should receive counseling regarding signs of pneumonitis initiation or worsening and point-of-care contact information. Modern RT approaches (e.g., VMAT/IMRT) should be prioritized where feasible. Amount of RT delivered should be minimized: ○ Lung V20 < 37% ○ MLD: <20 Gy For high-risk patients, consider whether alternative strategies are warranted (e.g., induction chemotherapy). Direct communication between RO and MO is encouraged for high-risk patients, as this may influence decision to initiate durvalumab consolidation therapy. Patients suspected of having ILD at baseline should be referred for pulmonary function tests and other risk assessments before initiation of cCRT ± durvalumab. Potentially avoid use of taxane chemotherapy in high-risk patients (mixed evidence ^b^).
P3. What approach is recommended to determine the etiology of symptomatic pneumonitis?	**Recommendation P3.1:** Presentation of pneumonitis during durvalumab consolidation therapy may reflect RP or IO-related pneumonitis. Patients should be assessed by the treating RO to help determine underlying etiology. *(Level of agreement: 8 agree, 1 disagree)* **Recommendation P3.2:** The RO should compare the radiation plan with changes on CT imaging. In RP, lung parenchymal changes generally conform to the radiation treatment field, while IO-related pneumonitis is more likely to present with bilateral or diffuse lung changes. *(Level of agreement: unanimous)*	Changes characteristic of RP are typically observed within the 20 Gy isodose line.
P4. What treatments are effective for management of RP?	**Recommendation P4.1:** Asymptomatic (grade 1) RP is common after cCRT and does not warrant investigation or treatment; however, increased monitoring may be warranted for patients presenting with new radiological changes after initiation of durvalumab consolidation therapy. *(Level of agreement: unanimous)* **Recommendation P4.2:** Patients with confirmed grade 2 RP should be followed under close observation. Prompt initiation of corticosteroid therapy should be considered in the event of worsening symptoms, as well as supplemental oxygen as clinically appropriate. Consider holding durvalumab if patient has initiated consolidation therapy. *(Level of agreement: unanimous)* **Recommendation P4.3:** Patients with confirmed grade 3/4 RP should promptly receive corticosteroid therapy and supplemental oxygen as clinically appropriate. Consider whether referral to respirology and/or hospitalization are warranted. Hold durvalumab if patient has initiated consolidation therapy. *(Level of agreement: unanimous)* **Recommendation P4.4:** Recommended corticosteroid therapy is oral prednisone 1 mg/kg/day up to 60 mg/day; treatment should be tapered slowly over a duration of at least 6 weeks once RP has clinically improved. Simultaneous initiation of PPI therapy is also recommended, as well as consideration of prophylaxis for PJP. *(Level of agreement: unanimous)*	Given risk of worsening associated with grade 2 RP, consider close follow-up every 1 to 2 weeks with monitoring of clinical symptoms and changes on chest imaging. Use of IV methylprednisolone may be preferable over prednisone for patients admitted to hospital. To decrease effects of corticosteroid therapy on bone, consider prophylactic treatment with calcium, vitamin D, and/or oral bisphosphonates. If initiated, PPI treatment duration should be limited to the corticosteroid treatment phase whenever possible. If held, durvalumab should only be resumed once prednisone therapy (or equivalent) is tapered to ≤10 mg/day (see Question P7).
P5. What treatments are effective for management of IO-related pneumonitis?	**Recommendation P5.1:** Patients with asymptomatic (grade 1) IO-related pneumonitis should receive more frequent follow-up with oxygen saturation and chest X-ray. Consider holding durvalumab on a case-by-case basis. *(Level of agreement: 8 agree, 1 abstains)* **Recommendation P5.2:** Patients with confirmed grade 2 IO-related pneumonitis should have durvalumab suspended, corticosteroid therapy promptly initiated, and supplemental oxygen provided as clinically appropriate. Monitor closely; if pneumonitis persists or worsens after 48 to 72 h, treat as grade 3 or 4. *(Level of agreement: unanimous)* **Recommendation P5.3:** Patients with confirmed grade 3 or 4 IO-related pneumonitis should have durvalumab suspended or discontinued and corticosteroid therapy promptly initiated. Supplemental oxygen, hospitalization, and respirology referral are warranted. If pneumonitis persists or worsens after 48 h, consider initiating a non-steroidal immunosuppressive agent. *(Level of agreement: unanimous)* **Recommendation P5.4:** Recommended outpatient corticosteroid therapy is oral prednisone 1–2 mg/kg/day; treatment should be tapered slowly over a duration of at least 6 weeks once pneumonitis has improved clinically. Simultaneous initiation of PPI therapy is recommended, as well as consideration of prophylaxis for PJP. *(Level of agreement: 8 agree, 1 abstains)*	If pneumonitis etiology is uncertain, a conservative approach including durvalumab hold and steroid initiation is recommended. If infection is suspected, consider referral to an infectious disease specialist and determine whether corticosteroid therapy can be initiated on a case-by-case basis. Use of IV methylprednisolone may be preferable over prednisone for patients admitted to hospital. To decrease effects of corticosteroid therapy on bone, consider prophylactic treatment with calcium, vitamin D, and/or oral bisphosphonates. If initiated, PPI treatment duration should be limited to the corticosteroid treatment phase when possible. For patients whose symptoms remain uncontrolled while on corticosteroid therapy, consider escalation of treatment management using infliximab or mycophenolate. A second dose of infliximab may be warranted if there is no improvement after 14 days.
P6. Which patients with RP are suitable for initiation of durvalumab consolidation?	**Recommendation P6.1:** Consider initiating durvalumab for patients with asymptomatic RP after cCRT; more frequent follow-up with chest X-ray may be warranted. *(Level of agreement: unanimous)*	Most patients will experience grade 1 RP after cCRT and are suitable for initiation of durvalumab consolidation. The typical time to onset of symptomatic RP is 2 to 6 months after cCRT completion; therefore, RP generally occurs after durvalumab has been initiated. Initiation of IO among patients with ongoing symptomatic RP is inadvisable. Follow-up frequency (including chest X-ray imaging) should be individualized in accordance with patients’ risk of deterioration and/or hospitalization. Consider monthly or Q1–2W visits for higher-risk cases.
P7. Which patients are suitable for durvalumab re-initiation after resolution of pneumonitis?	**Recommendation P7.1:** For patients with confirmed RP who have resolution of symptoms and corticosteroids tapered to ≤10 mg/day, consider re-initiating durvalumab. *(Level of agreement: unanimous)* **Recommendation P7.2:** For patients with IO-related pneumonitis who have symptom resolution and corticosteroids tapered to ≤10 mg/day, the decision to re-initiate durvalumab should be individualized on the basis of patient characteristics and shared decision-making. *(Level of agreement: 8 agree, 1 disagree)*	Considerations relevant to decision-making regarding durvalumab re-initiation may include patient preference, age, PD-L1 expression, and proximity to the treating care center. Pneumonitis grade should also be considered before restarting durvalumab. Re-initiation is not generally recommended for patients with grade 3 or 4 events but may be appropriate for grade 3 pneumonitis that promptly and completely responds to corticosteroid therapy. If symptomatic pneumonitis returns upon re-initiation of durvalumab, the IO should be permanently discontinued.
P8. Which HCPs should be involved in the care of patients who experience pneumonitis?	**Recommendation P8.1:** Multidisciplinary team care is essential for optimizing follow-up of pneumonitis and must involve shared responsibilities, clear communication, and collaboration. *(Level of agreement: 8 agree, 1 disagree)* **Recommendation P8.2:** Radiation and medical oncologists should communicate regarding patient status and classification of pneumonitis etiology. If RP, the RO should be responsible for management and follow-up until resolution; if IO-related, the MO should be responsible. *(Level of agreement: unanimous)*	MDT may include the RO, MO, nurse, NP, respirologist, radiologist, pharmacist, PCP, and/or infectious disease specialist. For a proportion of patients, the etiology of pneumonitis will remain uncertain. In such cases, sharing of care responsibilities between the MO and RO is recommended. PCP should receive ongoing documentation regarding patient management approach and status.

^a^ V20 defined as total lung volume minus GTV in studies of recommended values. ^b^ Meta-analysis/larger studies have reported a significant effect of taxane therapy on pneumonitis risk. cCRT, concurrent chemoradiation therapy; CT, computed tomography; GTV, gross tumor volume; HCP, healthcare professional; ILD, interstitial lung disease; IMRT, intensity-modulated radiation therapy; IO, immuno-oncology therapy; IV, intravenous; MDT, multidisciplinary team; MLD, mean lung dose; MO, medical oncologist; PCP, primary care practitioner; PD-L1, programmed cell death ligand 1; PJP, pneumocystis jiroveci pneumonia; PPI, proton pump inhibitor; QXW, every X weeks; RO, radiation oncologist; RP, radiation pneumonitis; VMAT, volumetric modulated arc therapy.

## Data Availability

No new data were created or analyzed in this study. Data sharing is not applicable.

## Supplementary Materials

The following supporting information can be downloaded at https://www.mdpi.com/article/10.3390/curroncol31110483/s1. File S1: Additional details on study methods and “magic mouthwash” formulations.
