# Supplementary material for: Esophagitis and Pneumonitis Related to Concurrent Chemoradiation ± Durvalumab Consolidation in Unresectable Stage III Non-Small-Cell Lung Cancer: Risk Assessment and Management Recommendations Based on a Modified Delphi Process
Source: Curr Oncol. 2024 Oct 23;31(11):6512–35. doi: 10.3390/curroncol31110483 (PMC11593044; doi:10.3390/curroncol31110483)
Supplement: Supplementary file 1 [file curroncol-31-00483-s001.zip › curroncol-3233382-supplementary.pdf]

SUPPLEMENTARY MATERIALS

Esophagitis and Pneumonitis Related to Concurrent Chemoradiation ± Durvalumab Consolidation in Unresectable Stage III Non-small Cell Lung Cancer: Risk Assessment and Management Recommendations based on a Modified Delphi Process

Anthony M. Brade*, Houda Bahig, Andrea Bezjak, Rosalyn A. Juergens, Charmaine Lynden, Nicolas Marcoux, Barbara Melosky, Devin Schellenberg, Stephanie Snow

Literature Review, Data Synthesis, & Preliminary Clinical Questions

To inform development of clinical questions and recommendations for the EWG, a targeted literature review was conducted using PubMed. Search syntax included combinations of the following terms: non-small cell lung cancer, esophagitis, pneumonitis, chemotherapy, radiation, chemoradiation, and chemoradiotherapy. The search time horizon spanned Jan 1, 2008, through March 21, 2023, and limits were applied to focus the results. More than 440 articles were considered for relevance to the research topic; bibliographies of selected publications were also reviewed. Selected studies were synthesized in Microsoft Excel and the following information was extracted for esophagitis and pneumonitis, when reported: study design, patient population, signs and symptoms, time to onset, incidence rates, risk factors, assessment, risk mitigation, treatment, and initiation and re-initiation of immuno-oncology therapy.

Based on the results of the literature review and clinical experience, one healthcare professional (A.M.B.) drafted 10 preliminary questions (4 for esophagitis and 6 for pneumonitis) that encompassed four major themes: risk factors, risk mitigation, management, and care team involvement. In October 2023, the draft questions and summarized findings from the literature review were provided to the EWG.

Expert Working Group Meeting

In November 2023, the expert working group (EWG) convened virtually via video teleconference. The experts provided feedback on the preliminary clinical questions and discussed potential recommendations and other pertinent insights. All comments were captured in a summary report, which informed revision of the questions and drafting of recommendations and related considerations and caveats. Twelve updated clinical questions and 29 draft recommendations were developed for EWG voting and feedback.

Modified Delphi Process

Between December 2023 and March 2024, a modified Delphi process was undertaken. During each round, EWG members indicated their agreement with each recommendation by selecting “agree”, “disagree”, or “abstain” (i.e., if a topic was considered outside the expert’s scope); free-text commentary was also provided. After each round, voting/feedback was compiled by a medical writer and anonymized results were reviewed by AMB. A response level of 100% was required before moving to the next round. Recommendations with potential for refinement and/or with consensus <75% were revised by AMB; key considerations were simultaneously edited. Other EWG members were blinded to the process of statement

modification. The process was repeated until consensus was achieved; where applicable, reasons for disagreement were documented. A total of three rounds were conducted.

Risk Assessment Profiles

Using feedback gathered during the modified Delphi process, preliminary patient risk profiles were developed for esophagitis and pneumonitis to further support patient assessment and management. Based on further input from the EWG and in alignment with the final consensus recommendations, figures were created for each adverse event, with considerations stratified according to higher, moderate, or lower risk.

Table S1. Examples of "Magic Mouthwash" formulations

Province	Center	Description	
Vancouver	BC Cancer – Vancouver Centre	**Magic Mouthwash**	Qty: 1,000 mL
		Diphenhydramine HCl Elixir (12.5 mg/5 mL)	300 mL
		Nystatin suspension (100,000 units/mL)	100 mL
		Hydrocortisone (100 mg/2 mL)	2 mL
		Distilled water	Add to make 1,000 mL
		**Directions:**<br>□ 20 mL swish and swallow QID<br>□ 20 mL swish and spit QID	
Ontario	Juravinski Cancer Centre	**TozEllis Mouthwash**	
		Diphenhydramine capsule	550 mg
		Nystatin suspension	50 mL
		Hydrocortisone injection	50 mg
		Tetracycline capsule	500 mg
		Sterile water	QS to 250 mL
		15-30 mL Q2-4h pm<br>Swish in mouth for 1-2 minutes then spit out or swallow if directed.	
		**The Wright Mouthwash**	
		Diphenhydramine capsule	375 mg
		Nystatin suspension	50 mL
		Dexamethasone injection	2.8 mg
		Sterile water	QS to 250 mL
		5 mL QID<br>Swish in mouth for 1-2 minutes then spit out or swallow if directed.	
Québec	Centre Hospitalier de l'Université de Montréal	**Rince-Bouche Magique**	Qté: 390 mL
		Diphenhydramine 2.5 mg/mL (PMS-diphenhydramine en solution orale)	120 mL
		Nystatine 100 000 unités/mL (suspension orale)	30 mL
		Hydrocortisone (comprimés) ou hydrocortisone en poudre	50 mg
		Eau distillée	240 mL
		**Posologie:** 12 mL po QID (gargariser et avaler), 5 à 10 min avant chaque repas et au coucher. Service 500 mL à la fois.	

	CHU de Québec	**Gargarisme de base (en présence d'une mucosite de grades 2 ou 3):**<br>15 mL en gargarisme QID jusqu'à résolution des symptôms. Peut-être avalé si lésions profondes.<br><br>Diphenhydramine (Benadryl^{MD}) 12,5 mg/5 mL (DIN 99113920)<br>Nystatine suspension 100 000 unités/mL (DIN 00792667)<br>Hydrocortisone (Cortef^{MD}) 20 mg/comprimé (DIN 00030929)<br>Eau distillée (DIN 00906719)<br>Hydroxyde de magnésium/aluminium (DIN 99002574)<br><br>**Stabilité:** 30 jours au réfrigérateur	Qté: 240 mL<br><br><br>300 mg = 120 mL<br>3 000 000 unités = 30 mL<br>50 mg = 2 comprimés et ½<br>25 mL<br>Compléter jusqu'à 240 mL
		**Gargarisme avec lidocaïne (en présence d'une mucosite de grades 2 ou 3 avec une douleur importante):**<br>15 mL en gargarisme QID jusqu'à résolution des symptôms. Peut-être avalé si lésions profondes.<br><br>Lidocaïne visqueuse 2% (Xylocaïne^{MD}) (DIN 01968823)<br>Sucralfate suspension 1 mg/5 mL (DIN 02103567)<br>Nystatine suspension 100 000 unités/mL (DIN 00792667)<br>Eau distillée (DIN 00906719)<br><br>**Stabilité:** 30 jours au réfrigérateur	Qté: 250 mL<br><br><br>500 mg = 25 mL<br>5 g = 25 mL<br>2 400 000 unités = 24 mL<br>Compléter jusqu'à 250 mL
Nova Scotia	Queen Elizabeth II Health Sciences Centre	**Magic Mouthwash**<br><br>Diphenhydramine syrup<br>Nystatin oral suspension<br>Lidocaine 2% viscous<br>Kaopectate<br><br>**SIG:** 5 to 10 mL, Q4-6h PRN<br>Swish and spit / Mitte: 200 mL Refill x3	Qty: 200 mL<br><br>50 mL<br>50 mL<br>50 mL<br>50 mL
		**BDL Mouthwash**<br><br>Benadryl<br>Lidocaine, 2%<br>Dioval<br><br>SIG: Use 10 mL QID as directed<br>Mitte: 300 mL<br>Repeat x5	Qty: 300 mL<br><br>150 mL<br>50 mL<br>50 mL
